# Dynamic Involvement of Telocytes in Modulating Multiple Signaling Pathways in Cardiac Cytoarchitecture

**DOI:** 10.3390/ijms23105769

**Published:** 2022-05-21

**Authors:** Ioana Cucu, Mihnea Ioan Nicolescu, Ștefan-Sebastian Busnatu, Cătălin Gabriel Manole

**Affiliations:** 1 Faculty of Medicine, “Carol Davila” University of Medicine and Pharmacy, 050474 Bucharest, Romania; ioana.cucu@stud.umfcd.ro; 2 Division of Histology, Faculty of Dental Medicine, “Carol Davila” University of Medicine and Pharmacy, 050474 Bucharest, Romania; 3 Laboratory of Radiobiology, “Victor Babeș” National Institute of Pathology, 050096 Bucharest, Romania; 4 Department of Cardiology-“Bagdasar Arseni” Emergency Clinical Hospital, Faculty of Medicine, “Carol Davila” University of Medicine and Pharmacy, 041915 Bucharest, Romania; 5 Department of Cellular & Molecular Biology and Histology, Faculty of Medicine, “Carol Davila” University of Medicine and Pharmacy, 050474 Bucharest, Romania; catalin.manole@umfcd.ro; 6 Laboratory of Ultrastructural Pathology, “Victor Babeș” National Institute of Pathology, 050096 Bucharest, Romania

**Keywords:** telocytes, heart failure, WNT, TGFB, PI3K/AKT

## Abstract

Cardiac interstitium is a complex and dynamic environment, vital for normal cardiac structure and function. Telocytes are active cellular players in regulating main events that feature myocardial homeostasis and orchestrating its involvement in heart pathology. Despite the great amount of data suggesting (microscopically, proteomically, genetically, etc.) the implications of telocytes in the different physiological and reparatory/regenerative processes of the heart, understanding their involvement in realizing the heart’s mature cytoarchitecture is still at its dawn. Our scrutiny of the recent literature gave clearer insights into the implications of telocytes in the WNT signaling pathway, but also TGFB and PI3K/AKT pathways that, inter alia, conduct cardiomyocytes differentiation, maturation and final integration into heart adult architecture. These data also strengthen evidence for telocytes as promising candidates for cellular therapies in various heart pathologies.

## 1. Introduction

The heart is frequently perceived as a mere blood muscular pump. However, previous studies have demonstrated that cardiomyocytes (CMs) represent about 75% (only) of the heart volume. We may conclude that, in terms of volumes, the myocardial interstitium is surprisingly well represented. Moreover, myocardial interstitium is more than a passive entity, but rather a complex and dynamic environment. It represents an important structural and signaling system within the myocardium, being vital for normal cardiac structure and function [1]. As an example, the progression of the heart failure (HF) in terms of (ultra)structural changes (from myocardial hypertrophy to morphopathologically established HF with clinical outcome) seems to be orchestrated by myocardial interstitium, also. In terms of lesion progression, this means an advancement from the initial interstitial edema (which impair the subendocardic coronary perfusion), to later interstitial fibrosis (associated with the decline of the ventricular systolic function, abnormal cardiac remodeling, and increased ventricular wall rigidity) [2,3]. Regional myocardial fibrosis as a result of ischemic heart disease is well described as resulting in the progressive deterioration of myocardial function.

## 2. Cardiac Telocytes 

Telocytes (TCs), a novel distinct type of interstitial cell, have been reported in the last decade within all three layers of the normal human and mammalian heart [4,5,6,7,8,9]. The presence of TCs was also reported within cardiac stem cell (CSCs) niches, where they seem to participate in the process of cardiac renewing/regeneration [10]. They were termed “Telocytes” considering the ultrastructural features of their cellular prolongations. Their prolongations were termed telopodes, and they are typically present in an alternation of thin segments (podomers) and dilated segments (podoms) [11]. Podoms accommodate mitochondria, endoplasmic reticulum and caveolae; a combination of organelles characteristic for their so-called “calcium uptake/releasing units”. For the characterization of heart TCs numerous methods have been used, including transmission electron microscopy (TEM), scanning electron microscopy, electron tomography, Focus Ion Beam—Scanning Electron Microscopy, immunofluorescence, immunohistochemistry (IHC), miRNA profile, and genomics and proteomics analyses [12,13,14,15,16,17,18]. However, light microscopy, due to its limitations, cannot give an accurate positive diagnosis for TCs. Other cellular elements in the cardiac interstitium may be accidentally mistaken with TCs, e.g., endocardial tip cells [19]. Additionally, for a better understanding of TCs function(s), their positive expression to a set of markers was tested: CD34, c-kit/CD 117, S-100, desmin, vimentin, Alpha-smooth muscle actin (a-SMA), etc. Unfortunately, the results of IHC are still equivoque. TCs showed different positivity to the tested markers, depending on the organ and animal species used. Howbeit, the most reliable combination of markers for TCs remains the combination of CD34/Platelet-derived growth factor receptor (PDGFR). Previous studies showed that in cell culture, TCs secrete VEGF, cytokines and chemokines. This was demonstrated by SELDI-TOF mass spectrophotometry and xMAP Technology (Luminex). RTqPCR demonstrated that TCs differentially express miR-193, compared with other cells, suggesting that TCs represent a different type of interstitial cells. Moreover, by this method, it was shown that TCs also express measurable quantities of pro- angiogenic microRNAs (let-7e, 10a, 21, 27b, 100, 126-3p, 130a, 143, 155, 503). The abovementioned indicates the involvement of TCs in neo-angiogenesis [20].

In HF, extracellular matrix remodeling represents an important component of myocardial remodeling. Extracellular matrix remodeling is associated with specific Matrix Metalloproteinases/Tissue inhibitors of metalloproteinases (MMPs/TIMPs) balance. For example, in the later stages of myocardial infarction (MI), in its progression to HF, interstitium could show cartilaginous and osseous metaplasia, this being detrimental to its normal architecture and also to TCs presence within [21]. The increased amounts of collagen from interstitial fibrosis are associated with apoptosis of TCs and shrinking of their telopodes. Moreover, TCs were found to be almost completely absent in fibrotic areas of myocardium affected by systemic sclerosis [22].

## 3. Network of Interactions between Signaling Pathways

CMs plasticity has emerged as a critical blueprint to induce heart regeneration and myocardial remodeling. Embryonic/fetal and neonatal CMs undergo resolute proliferation, aiming to adapt to increased circulation workload [23]. Adult CMs have withdrawn from the cell cycle as they have been considered as terminally differentiated cells and have a moderate capacity to proliferate [24]. The assimilation of several signaling pathways between CMs and TCs, including WNT, transforming growth factor beta (TGFB), phosphatidylinositol 3-kinase/v-akt murine thymoma viral oncogene homolog and protein kinase B (PI3K/AKT), in concert with transcription factors (TFs), represents a key step to procure regenerative potential by prompting CMs to re-enter the cell cycle and reprogram to a fetal-like state. Below, we delineate a global landscape of protein–protein interaction networks and putative therapeutic approaches that might promote cardiac repair and bolster cardioprotective effects after myocardial injury, highlighting the significance of these signaling pathways in normal cardiac development and the pathogenesis of heart diseases.

### 3.1. WNT Signaling Pathway

The WNT pathway is an indispensable regulator in cardiogenesis, cardiac remodeling, adult homeostasis, tissue, and organ size, cell survival and apoptosis, and is also involved in elaborating cardiomyopathies and wound healing after MI and HF [25,26,27]. WNT signaling comprises substantial pathways: a canonical pathway (WNT/beta-catenin) and a non-canonical pathway, which is further subdivided into the WNT/Ca^2+^ and planar cell polarity (PCP) pathways [28]. Regarding the canonical pathway, in the WNT-off state, the cytoplasmic beta-catenin protein is persistently degraded by a complex of proteins (the “destruction complex”), comprising Adenomatous polyposis coli (APC), Casein kinase 1 (CK1), Glycogen synthase kinase 3 beta (GSK3B), and the scaffolding protein Axin [29,30]. Cooperatively, these proteins prompt post-translational modifications (PTMs), resulting in a phosphorylated beta-catenin, which is acknowledged by Beta transducin repeats containing protein (BetaTrCP) and targeted for ubiquitination and proteasomal degradation [31]. Synchronously, these circumstances avoid beta-catenin translocation to the nucleus, and WNT/beta-catenin target genes are consequently suppressed by the T cell factor/Lymphoid enhancer factor (TCF/LEF) family of proteins [30,32,33,34]. In the WNT-on state, a WNT ligand attaches to a Frizzled (FZD) receptor, linked to its coreceptor, the Lipoprotein receptor-related protein 5/6 (LRP5/6) [30,35]. Upon the recruitment of the scaffolding protein Disheveled (DVL), the WNT–FZD–LRP complex generates the employment of the Axin complex to the receptors, leading to the inhibition of beta-catenin phosphorylation, and thereby to the stabilization of beta-catenin, which shifts into the nucleus, establishes a composite with TCF/LEF and activates WNT-target genes, including *cyclin-D*, vascular endothelial growth factor (*VEGF*), *survivin*, and *c-Myc* [30,36,37,38]. Moreover, the Leucine-rich repeat-containing G-protein coupled receptor (LGR5) also represents a target of WNT signaling and hallmarks normal SCs in multiple tissues, inclusive of the heart [39,40,41]. LGR5 interacts with R-Spondins (RSPOs) and proceeds in partnership with FZD and LRP5/6 to enhance WNT/beta-catenin and WNT/PCP signaling [42,43]. Evolutionarily conserved inhibitors and activators regulate WNT signaling. Activators of WNT signaling incorporate WNT ligands, Norrin, and RSPOs [44]. WNT inhibitors appertain to small protein families, comprising Dickkopf proteins (DKKs), secreted Frizzled-related proteins (sFRPs), WNT-inhibitory factor 1 (WIF-1), Wise/SOST, Cerberus, Insulin-like growth factor binding protein 4 (IGFBP-4), Shisa, WNT-activated inhibitory factor 1 (WAIF1/5T4), adenomatosis polyposis coli down-regulated 1 (APCDD1), and TIKI1 [45]. They antagonize WNT signaling by prohibiting ligand-receptor interconnection [45]. Intriguingly, LGR5/RSPO complex is masterly to counterbalance Ring finger protein 43 (RNF43) and Zinc and ring finger protein 3 (ZNRF3) transmembrane E3 ligases [46]. These enzymes constitute WNT targets that expel WNT receptors from the cell surface, therefore coordinating WNT signaling output. Notably, Sahara et al. [41] disclosed for the first time the existence of a subset of cono-ventricular region LGR5^+^ cardiac progenitor cells (CPCs), which emerge characteristically within the outflow tract (OFT) of human hearts and bolster cardiogenesis through the expansion of the mesodermal second heart field (SHF) progenitors PDGFR-alpha^+^ ISL1^+^ (Islet 1/Insulin gene enhancer 1) TNNT2^+^ (Troponin T2), intermediating favorably into CMs. Interestingly, Halpern et al. ascertained a decidedly conserved population of LGR5^+^ TCs within the niche of the intestinal villus tip [47]. These TCs also constitute a source of RSPO3 and canonical WNT morphogens to orchestrate the gene expression instruction [47]. Similarly, another recent study disclosed that FOXL1^+^ TCs, a subset of PDGFR-alpha^+^ cells, also express LGR5, RSPO3, Sonic hedgehog (SHH), Bone morphogenetic protein 4 (BMP4), canonical and non-canonical WNT morphogens, DKK2, DKK3, ZNRF3 and sFRP1 [47,48]. Likewise, RSPO3 represents a crucial modulator, indispensable for cardiogenesis, which is enriched within the ISL1^+^ cono-ventricular progenitors [41]. Furthermore, the assignment of LGR5 in the differentiation of human pluripotent SCs (hPSCs) into CMs should be underscored: the upregulation of *LGR5* during the prompt stage of CMs differentiation advocates the nuclear translocation of beta-catenin to the nucleus to assert the target genes, bolstering cardiomyogenesis [49]. A paramount approach that will portray TCs immunophenotype in terms of molecular signals within zonated organs is loss-of-function study, either using gene ablation archetypes or ablating TCs in vivo in conjunction with transmission electron microscopy (TEM). 

**Figure 1:**
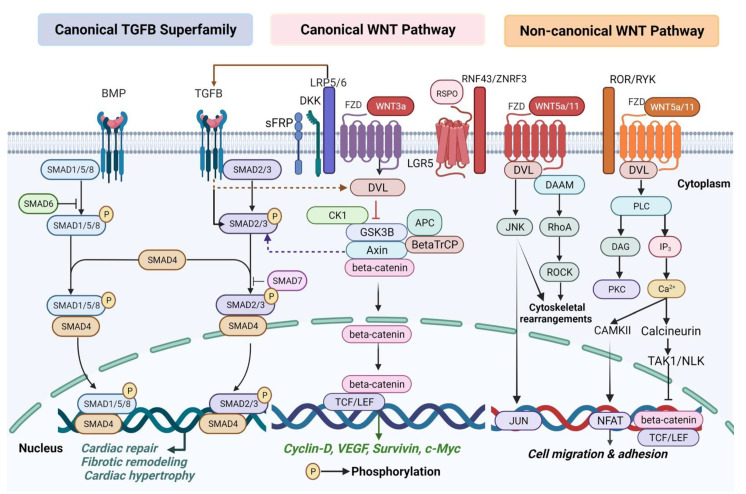
An illustrative representation of the interlinking networks between WNT and TGFB signaling pathways. In the sketch are delineated the intricate protein–protein interaction latticeworks within cardiomyocytes and telocytes, showing a complementary synergism to induce cardiomyogenesis, cardiac regeneration, and pathogenesis of heart diseases. The figure is widely scrutinized in the main text. The black arrows designate main signaling pathways; the dashed arrows indicate activation; the continuous blunt-ended arrows pinpoint inhibition. APC: Adenomatous polyposis coli; BetaTrCP: Beta transducin repeats-containing protein; BMP: Bone morphogenetic protein; CAMKII: Calcium calmodulin-dependent protein kinase II; CK1: Casein kinase 1; DAAM: Disheveled-associated activator of morphogenesis; DAG: Diacylglycerol; DKK: Dickkopf; DVL: Dishevelled; FZD: Frizzled; GSK3B: Glycogen synthase kinase 3 beta; IP3: Inositol trisphosphate; JNK: c-Jun N terminal kinase; LGR5: Leucine-rich repeat-containing G-protein coupled receptor; LRP5/6: Lipoprotein receptor-related protein 5/6; NFAT: Nuclear factor of activated T-cells; PKC: Protein kinase C; PLC: Phospholipase C; RNF43: Ring finger protein 43; ROCK: Rho-associated protein kinase; RSPO: R-spondin; SMAD: Small mothers against decapentaplegic; TAK1/NLK: Transforming growth factor beta-activated kinase 1/Nemo like kinase; TCF/LEF: T cell factor/Lymphoid enhancer factor; TGFB: Transforming growth factor beta; sFRP: Secreted Frizzled-related proteins; ZNRF3: Zinc and ring finger protein 3. Created with https://biorender.com/, accessed on 30 April 2022.

The emblem of the non-canonical signaling pathway is its beta-catenin-autonomous exertions [50]. The DVL-c-Jun N terminal kinase (JNK) pathway/PCP pathway is required in cellular polarity [51]. Of the members of the WNT family of glycoproteins, WNT5a is one of the most eminently scrutinized non-canonical members. Once WNT interacts with FZD along DVL to assemble a complex with the Disheveled-associated activator of morphogenesis (DAAM), the JNK kinase pathway is activated, proceeding with cytoskeletal rearrangements [51]. In addition, DAAM propels RhoA, which further actuates Rho-associated protein kinase (ROCK) [51,52]. In the non-canonical WNT/Ca^2+^ signaling, the cooperation between the ROR/RYK co-receptor and WNT/FZD generates the setout of Diacylglycerol (DAG) and Inositol trisphosphate (IP3) [51,53,54]. DAG activates Protein kinase C (PKC) [54]. IP3 disperses through the cytosol to produce the release of Ca^2+^ from the endoplasmic reticulum, which subsequently activates the Calcium calmodulin-dependent protein kinase II (CaMKII) and the Ca^2+^-Calcineurin axis to stimulate the Nuclear factor of activated T-cells (NFAT) and Transforming growth factor beta-activated kinase 1/Nemo-like kinase (TAK1/NLK) [51,54]. Intriguingly, activated TAK1/NLK can impede TCF/beta-catenin signaling, while NFAT can synchronize migration and cell adhesion [55,56] (Figure 1).

#### 3.1.1. WNT Signaling in Cardiogenesis and Congenital Heart Diseases

The cardiogenesis constitutes a multifaceted operation, inaugurated by the specification of CPCs from the mesoderm [57]. The WNT signal transduction pathway turns out to substantially regulate the process of cardiogenesis. Subsequent to the specification of the mesoderm, the canonical WNT pathway is suppressed by WNT antagonists (BMP 2/4, SHH), WNT inhibitors (DKK1), along with non-canonical WNTs (WNT5a, WNT11), intending to downregulate pluripotency [57,58]. Notably, TCs exhibit pluripotency-related genes, aside from *c-kit*, including *NANOG*, *OCT4*, *KLF4* within murine lung tissue [59]. In this regard, it was demonstrated that TCs coordinate morphogenetic mechanisms of cardiac development to mastermind CPCs for actuating and commitment, and also TCs hold promise to generate mesenchymal stem cells (MSCs) [60,61]. Several TFs, comprising cardiac markers NK2 transcription factor related locus 5 (NKX2-5), GATA4/5/6, Myocyte enhancer factor 2C (MEF2C), TBX5/20, HAND1/2, and ISL1, contribute to the cardiogenic program to induce the primordial configuration of the heart [58,62]. 

Downregulation of *WNT11* is associated with cyanotic congenital heart defects in Tetralogy of Fallot (TOF) hypoxemic infants [63]. The intricacy of the picture is exacerbated by the involvement of TCs in the pathogenesis of TOF [64]. Contacts between TCs and Ki-67^+^ CPCs were identified within immature myocardium, as the tactical emplacement and the ultrastructural features advocated their engagement in paracrine synchronization and SCs differentiation [64]. NKX2-5 is a pivotal TF in cardiac looping and its mutation brings about both hypoplastic right ventricle syndrome (HRVS) and hypoplastic left heart syndrome (HLHS) in humans [62,65,66]. Disruption of MEF2C function results in the downregulation of *HAND1/2*, altering the cardiac differentiation [62]. Other studies showed that deletion of the *beta-catenin* using the MEF2C-Cre mouse line caused right ventricular hypoplasia, whilst enhanced *beta-catenin* expression within the ISL^+^ progenitors resulted in right ventricular hyperplasia [67,68]. A recent paper shows that *WNT11* null mice displayed ventricular septal defects (VSDs) and abnormalities, giving rise to double outlet right ventricle (DORV) [69].

#### 3.1.2. WNT Signaling in Cardiac Remodeling and Heart Failure

Given that CMs are permanently differentiated cells and have an exiguous proliferation rate when they encounter an enlarged workload or injury, CMs undergo hypertrophic growth [25,70]. These adjustments generate a reduction in ventricular performance, inducing HF [26]. Postnatal heart growth is ruled by the physiological hypertrophy of the CMs [71]. By contrast, cardiac hypertrophy is again prompted under pathological circumstances, such as myocardial ischemia/reperfusion (I/R) injury and MI [72]. Several studies revealed that WNT signaling is switched on in manifold disease mechanisms throughout the wound healing course subsequent to MI. Injection of WNT3a within the border zone of the MI proceeded in an expanded infarct area and decreased cardiac function in mice [73]. Additionally, in a study conducted by Kobayashi et al. [74] the global knockout (KO) of *sFRP2* resulted in reduced cardiac fibrosis and improved heart function following experimental MI. It is well described that the expression of sFRPs within the heart after experimental MI is favorable for cardiac remodeling [75,76]. In studies attributed to HF, an increased proportion of sFRPs has been pinpointed [25]. Decreased *beta-catenin* levels correspond to increased mRNA levels of sFRP3 and sFRP4 within human failing hearts [77]. Interestingly, sFRP3 and sFRP4 proportions were also found to be increased within the hearts of patients with both dilated cardiomyopathy (DCM) and coronary heart disease [77]. Askevold and colleagues [78] outlined as well that the increased extent of sFRP3 within the serum of HF patients was related to cardiovascular lethality. Moreover, upon mobilizing the canonical WNT signaling, within both human and murine failing hearts, the cumulation of beta-catenin within the nucleus led to increased expression of *c-Myc*, a gene asserted throughout cardiac stress [79]. Furthermore, adult CMs with double KO of *GSK3A* and *GSK3B* shows deleterious consequences on murine myocardium [80]. Double *GSK3* deletion is associated with mitotic catastrophe, ventricular dysfunction and immoderate cardiac remodeling, finally leading to lethal DCM [80]. Notably, WNT signaling has been documented to hand out to atrial fibrosis as a hallmark of architectural remodeling during atrial fibrillation [81]. WNT3a, except for WNT5a, has been revealed to switch on WNT/beta-catenin signaling by expressing the profibrotic target genes within cardiac fibroblasts [82]. Remarkably, in patients with distinct configurations of atrial fibrillation, accompanied by fibrosis and isolated atrial amyloidosis, TCs exhibited “transitional forms” with histiocytic function, and they were thought to be heavily involved in myocardial remodeling [83]. The synthetic exertion and proliferation of atrial TCs were delineated in response to myocardial hemodynamic overload [83]. Likewise, TCs were also involved in intercellular crosstalk in aortic valve calcification [84]. It was revealed that TC-derived extracellular vesicles (TC-EVs), carrying miR-30b, had inhibited the WNT/beta-catenin pathway, and had impeded osteogenesis and calcium deposition within valvular interstitial cells [84].

### 3.2. TGFB Signaling Pathway

A plethora of studies have delineated the significance of the TGFB superfamily signal transduction in the normal development and the pathogenesis of heart diseases, including cardiac repair, fibrotic remodeling and hypertrophy [85,86,87,88]. The TGFB superfamily can be subdivided into several families, comprising TGFBs (TGFB1, -B2, and -B3), the Bone morphogenetic proteins (BMPs), the Activins, Nodal, Inhibins, the Growth differentiation factors (GDFs), and anti-Mullerian hormone proteins [88]. Basically, TGFB and BMP ligands actuate serine/threonine kinases, classified as type I and type II receptors [89]. Ligands attach to type II receptors, resulting in conformational changes that prompt recruitment and complex generation with type I receptors [89]. Small mothers against decapentaplegic (SMAD) proteins are the inceptive responders which transduce the signal from the type I receptors [90,91]. SMADs include a family of proteins with distinct tasks: receptor-regulated (R-SMADs), inhibitory (I-SMADs), and common mediator (Co-SMADs) [89]. I-SMADs, SMAD6 and SMAD7, counteract the signaling interceded by R-SMADs and Co-SMAD [92]. Type I receptors activate R-SMADs: SMAD2 and SMAD3 in response to TGFB-like proteins, and SMAD1, SMAD5, and SMAD8 regarding BMPs [89,92]. Then, R-SMADs bind to Co-SMADs (SMAD4), and this composite shifts to the nucleus to regulate the responsive genes [88] (Figure 1). Conditional deletion of *BMP4* or BMP type I receptor (*BMPR1a*) under cardiomyogenic Cre drivers such as MESP1-Cre, NKX2- 5-Cre, or TNNT2-Cre mouse lines, gives rise to defective cardiac morphogenesis [93,94,95,96]. Furthermore, the single-cell RNA-sequencing (scRNA-seq) scrutiny wild type (WT) using MESP1- KO murine embryos has disclosed that *BMP4* could expressly mark the CM-committed population at E7.25 among MESP1^+^ mesodermal precursors [97,98]. Constitutive expression of TGFB is figured not only within both embryonic and adult CMs [88] but also within TCs [99,100]. Additionally, TGFB is conspicuously upregulated in models of experimental MI [86]. Activation of TGFB signaling seems to be primarily circumscribed within the infarct border zone and may induce extracellular matrix protein expression [86]. Regarding TGFB isoforms, TGFB1 is significantly upregulated after experimental MI and is also a leading cause of myocardial fibrosis and CM hypertrophy [101].

**Figure 2:**
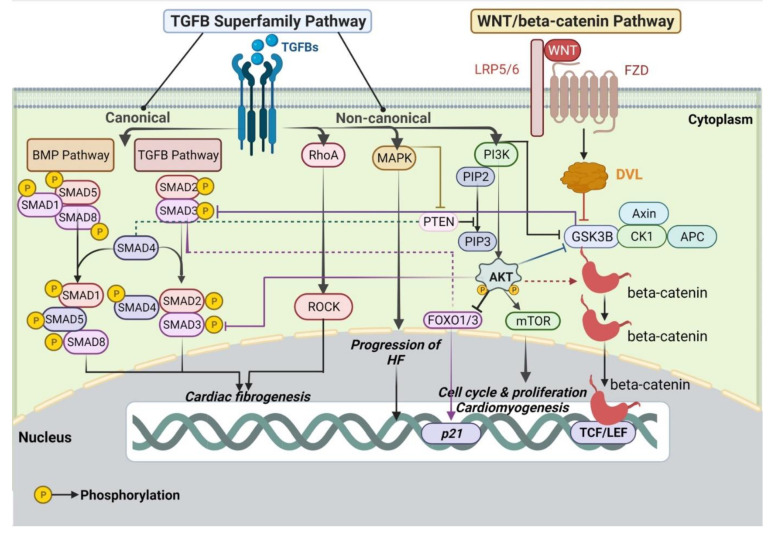
Illustration of the interconnecting networks between WNT, TGFB, PI3K/AKT/mTOR, and MAPK pathways. The figure is broadly discussed in the main text. The black arrows designate main signaling pathways; the dashed arrows indicate activation; the continuous blunt-ended arrows pinpoint inhibition. AKT: v-akt murine thymoma viral oncogene homolog/Protein kinase B; APC: Adenomatous polyposis coli; BMP: Bone morphogenetic protein; CK1: Casein kinase 1; FOXO1/3: Forkhead box proteins O1/3; FZD: Frizzled; GSK3B: Glycogen synthase kinase 3 beta; HF: heart failure; LRP5/6: Lipoprotein receptor-related protein 5/6; MAPK: Mitogen-activated protein kinase; mTOR: mammalian target of rapamycin; PI3K: phosphatidylinositol 3-kinase; PIP2: Phosphatidylinositol-4,5-bisphosphate; PIP3: Phosphatidylinositol-3,4,5-triphosphate; PTEN: Phosphatase and tensin homolog deleted on chromosome 10; ROCK: Rho-associated protein kinase; SMAD: Small mothers against decapentaplegic; TCF/LEF: T cell factor/Lymphoid enhancer factor; TGFB: Transforming growth factor beta. Created with https://biorender.com/, accessed on 30 April 2022.

TGFBs can induce diverse mechanisms which can be SMAD-dependent (canonical feedback) or SMAD-independent (non-canonical feedback). Several publications exhibit that the TGFB signaling moderates non-canonical signaling responses through the WNT, PI3K/AKT, Mitogen-activated protein kinase (MAPK) pathways [88,92,101,102,103] (Figure 2). Studies show that the substantial crosstalk between the TGFB and WNT pathways could be accountable for the transcription of pro-fibrotic genes. Cardiac fibrosis represents a pathological process corresponding to the development of HF [104]. In detail, these pathways could generate a positive/negative feedback loop that influences the transcriptional scheme of other signaling cascades. In bleomycin-induced lung fibrosis, loss of the WNT co-receptor *LRP5* reduced the expression of TGFB1 and declined the installation of fibrosis [105]. Additionally, another study provides evidence that Axin promoted SMAD3 attaching to the type I receptor to induce the tail-phosphorylation of SMAD3, activating the transcription of profibrotic genes [106]. A study conducted by Warner et al. [107] revealed that inducement of the TGFB pathway elicits an increment in DVL1 and SMAD3 binding in vivo. In response to SMAD-independent TGFB signaling, WNT/beta-catenin pathway has been disclosed to be activated by secretion of WNT proteins in inflammatory DCM [108,109]. Experiments have substantiated the significance of the RhoA-ROCK pathway in the adjustment of several cellular functions, notably in cardiac fibrogenesis [110,111]. It has been shown that CM-specific deletion of *RhoA* in response to chronic transverse aortic constriction (TAC) was associated with less fibrosis, greater dilation of ventricular walls, and potentiated HF [112]. Activation of the TGFB1/MAPK axis implies the progression of HF [101]. Within ischemic myocardial cells, MAPK and AKT are downregulated [113], and TGFB1 can activate MKK3/6 in the MAPK signaling pathway to withstand apoptosis in acute MI [101]. Interestingly, TGFB1 is interconnected with PI3K to regulate the cell cycle and proliferation of TCs [114]. Furthermore, Song et al. [99] investigated the mechanisms of the interplay between TGFB1 and PI3K isoforms regarding the modulation of lung TCs bio-behaviors: TGFB1 induced PI3Kp110 alpha, PI3K alpha/delta, PKC beta, or GSK3, in response to *ITGB1* deletion, bringing about the destabilization of TCs proliferation. Notably, a recent paper shows that in a model of induced unilateral ureteral obstruction (UUO), TCs attenuated renal fibrosis, reduced TGFB1 levels, and repressed SMAD2/3 phosphorylation in rats [115].

### 3.3. PI3K/AKT Signaling Pathway

A growing body of evidence delineates that the PI3K/AKT signaling pathway plays a key role in a multitude of processes related to the incidence, advancement, and pathological generation of cardiac fibrogenesis via the regulation of cell survival, apoptosis and cardiac contractility, albeit activating the target genes [100,116,117,118]. Essentially, the triggering of PI3K by receptor tyrosine kinases determines phosphorylation of phosphatidylinositol-4,5-bisphosphate (PIP2) to bring about phosphatidylinositol-3,4,5-triphosphate (PIP3) and activates the signaling pathway [119]. The activity of PI3K is counteracted by the tumor suppressor protein Phosphatase and tensin homolog deleted on chromosome 10 (PTEN), which detaches the phosphate group from the 3′ end of the inositol ring of PIP3, thereby negatively regulating the pathway [119,120]. AKT kinase represents activated downstream of PIP3 and synchronizes miscellaneous downstream effectors, including the mammalian target of rapamycin (mTOR), GSK3B, and Forkhead box proteins O1/3 (FOXO1/3) [118,121]. Suppression of mTOR with rapamycin avoids cardiac hypertrophy, advocating that mTOR plays a pivotal role in cardioprotection [122]. Along these same lines, AKT and mTOR represent critical players in angiogenesis throughout physiological cardiac hypertrophy, proposing that the dislocation of these molecules may induce pathological hypertrophy [27,123,124]. On one hand, a study conducted by Naito et al. [103] delineated the interconnection between PI3K and the canonical WNT pathways during early cardiomyogenesis (Figure 2). The PI3K/AKT pathway proceeds as a survival ingredient for the cardiac mesodermal cells during WNT-induced CM differentiation, aside from maintaining canonical WNT throughout CPCs commitment [103]. On the other hand, AKT phosphorylates and impedes the activity of GSK3, thus diminishing pathological cardiac interstitial fibrosis [118]. Guo et al. [125] evinced that GSK3B-KO induces SMAD3 hyperactivation, generating cardiac dysfunction within the ischemic heart and unfavorable fibrotic remodeling. Activated SMAD3 is associated with the FOXO1/3 TFs and they are simultaneously enrolled to the promoter of the growth inhibitory gene *p21* [126]. The PI3K/AKT activity debilitates SMAD3 through the AKT-mediated phosphorylation of FOXO TFs that are requisite for SMAD3 function [120]. Inhibition of the PI3K/AKT signaling activated GSK3B and the breakdown of beta-catenin via the suppression of the WNT/beta-catenin pathway [103]. Adding to this puzzle, TGFB enhances AKT phosphorylation via PI3K stimulation, thus disabling GSK3B, inducing cardiac fibrosis [127]. Interestingly, the amendment of PTEN function constitutes another passage for TGFB/BMP signaling to impact AKT activity. TGFB has been demonstrated to downregulate *PTEN* in the *SMAD4* null archetype, which appears to reckon on the promoting scheme of the MAPK pathway [128]. Likewise, another study indicated that *PI3K* deficiency safeguards against isoproterenol-induced HF and suggested that p110 gamma mediates pathological cardiac hypertrophy [129]. Notably, intraperitoneal administration of TCs improved the hardness of lung edemas associated with alterations in the mRNA expression of *MTOR*, *HIF1A*, *VEGF*, and *MMP9* within TCs [100]. TCs could increase airway epithelia proliferation and differentiation through driven mediators and exosomes to compensate for experimental acute lung injury [100]. Further strategies to define the assignment of TCs in cardiomyogenesis, cardiac remodeling, and pathogenesis of heart diseases include extensive scrutiny of their gene expression profiles and cell lineage trajectory and will open an avenue in the disclosure of novel “smart” drugs to target CMs regeneration, based upon understanding context-specific molecular mechanisms. However, in order to develop new and effective therapies that might include TC (i.e. for acute ischemic stroke), one must consider investigating also the mechanisms underlying brain damage and neuroprotection [130].

## 4. Telocytes Orchestration of Cell-to-Cell Communication to Integrate Molecular Signals

TCs have long been documented to be crucial modulators of intercellular signaling, either through direct contacts (point contacts, nanocontacts, and planar contacts) with CMs and adjacent nervous/vascular/immune elements or at long remoteness by releasing EVs and harmonizing SCs fate (proliferation, activation, differentiation) [131,132,133]. TCs possess intricate patterns of networking that might be regarded as the epicenter of the CSC niche modulation, playing a key role in mechanical sensing regulation [134]. Given the heterogeneous molecular profiles of TCs, it is crucial to understand the specific role of TCs subpopulations in regard to signal throughput implementation. The signaling pathways within TCs that bring about cardiac remodeling have been pinpointed in the shape of a protein regulatory latticework between TCs and CMs. In essence, TCs might be equipped with a “paraphernalia” of morphogens that act in a coordinated manner to harmonize the pathophysiological heart conditions. Remarkably, the safeguarding effect of TCs on fibrosis in diverse organs proposes these cells as master regulators in the pathogenesis of cardiac fibrosis [115]. It has been revealed that the amount of TCs markedly increased within the border zone after MI [20]. Interestingly, high expression of the downstream effectors of TGFB signaling is illustrated within the healing infarct [86]. In connection with the fact that SMAD2, -3, and -4 protein expression is markedly upregulated within the scar and border sector [135], in UUO, TCs have been shown to inhibit SMAD2/3 activation, thus suppressing the transcription of target genes related to fibrosis [115]. Moreover, several studies have disclosed the crosstalk between TGFB and WNT signaling cascades, not only in the pathogenesis of cardiac fibrosis but also in CMs regeneration. The administration of exogenous canonical WNT3a could induce cardiac fibrotic processes by stimulating TGFB production and activating SMADs [136]. Moreover, WNT3a attenuates CMs regeneration into the border zone of the infarct field [25]. Attractively, Halpern and co-workers [47] have validated the spatial TCs archetypes by using scRNA-seq quantifications: LGR5^+^ villus tip TCs (VTTs) express both canonical WNT3a and raised levels of the non-canonical WNT5a. Accordingly, loss of *WNT5a* generates a substantial reduction in SHF progenitors within the developing heart and is associated with an increase in WNT/beta-catenin signaling [137]. Further studies along these lines are required to elucidate the mechanism that connects the evolutive stages of TCs, their immunophenotypes from embryonic-fetal to adult life, with molecular cues expression that mediates CMs fate plasticity. Notwithstanding, the PI3K/AKT pathway constitutes a crucial regulator of cell proliferation and differentiation, and secures CMs from ischemic and hypoxic apoptosis [138]. TCs could preside over CMs repair and recovery from injury since they possess a potent capability of proliferation via the counteractive signaling mechanisms after TGFB1 treatment to inhibit PI3K/AKT- PKC- GSK pathways [99]. Ultimately, to better characterize TCs as distinctive cell types which govern the entire cardiac molecular landscape, prospective studies are needed to acknowledge their transcriptomic profiles using scRNA-seq technologies to portray the stromal gene expression signatures, as a consequence of crosstalk with CSCs/CPCs/CMs.

## 5. Putative Future Cellular Therapies

There are still clinical queries in cardiac pathology, especially regarding the existence of new biomarkers, or new optimal imaging modalities and diagnostic procedures that can better suggest or influence clinical decisions [139]. There are still difficulties that hinder the development of new cardiologic therapies in MI and HF. Inter alia, they especially refer to the high-resolution phenotyping of patients with specific cardiac disorders. Moreover, experimental models designated for specific cardiologic pathologies can closely mimic the etiology, physiopathology and morphopathology. Meantime, this could be a solid argument and choice for testing various experimental treatments, involving new molecules or cells and establishing their advantages for a given cardiac pathology, including HF of various etiologies [140]. Despite the optimizations and standardizations (of the last few years) for medical and interventional treatment for acute cardiac conditions (e.g., MI), the morbidity and mortality rates are still high [141].Therefore, finding new methods of cellular therapy based on TCs is a very attractive direction of research [142].

Recent past research and current research has focused on preclinical and clinical studies of regenerative therapies based on skeletal myoblasts, bone marrow stem and CSCs [143]. However, those foreseen good results mentioned almost every time are still far to be achieved. Thus, considering the present published data on TCs presence in normal heart and their cooperation with a great diversity of structural elements, it will be worthily to investigate and assess it focusing on heart regeneration/repair processes in cardiologic conditions. Perhaps, either for the classic stem cells therapy for diverse heart conditions, or for the next designed protocols that will include stem cell delivery in cardiac tissue (as a part of a complex cellular therapy), instead of using CSCs single-cell-therapy, a tandem TCs-CSCs should be considered more promising, as it is microscopically realistic.

Cardiac regeneration, specifically the process of CMs replacement and integration into normalizing cardiac architecture is regulated, among others, by few signaling pathways (WNT, TGFβ, PI3K/AKT). It is well acknowledged that downregulation to inhibition of WNT pathway reduces the mortality after MI and have roles in cardiac regeneration repair, since it can also influence vasculogenic progenitor cells [75]. These should be considered along the generally accepted implication of TCs in (neo)angiogenesis processes in affected hearts. On the other hand, it was shown that during the early cardiogenesis the upregulation of WNT signaling pathway amplify the precursor cells pool with evident implication in heart fields development and heart tube formation [96]. Additionally, the participation of TCs to cardiac stem cell niches was previously proved. Within this particular microenvironments TCs are acting as nursing cells for CMs progenitors, directing their normal incorporation into adult myocardial cytoarchitecture [144].

On the other hand, the SMAD-dependent pathways or non-SMAD pathways may mediate the TGFβ pathway, the latest being involved in myocardial repair/regeneration/remodeling processes. TGFβ pathway becomes activated in MI by still less known triggers [88]. However, the activation of the TGFb pathway stimulates the fibrotic process and matrix protein production that dominates the later stages of MI resolution [145]. In zebra fish hearts (well known for heart regeneration capacity), TGFβ was proved to be involved in endogenous cardiac regeneration [146]. Moreover, it is worth mentioning that zebrafish hearts (like newt hearts) are abundant in TCs, at this level by their long and slender telopodes TCs creating trabecula-like volumes in which they are establishing multiple contacts with CMs [8]. Noteworthy, in zebrafish hearts, cryoinjury of myocardium in TGFβ inhibited specimens abolish heart regeneration [147]. Therefore, finding ways of leveraging (at least members of) this TGFβ superfamily and also TCs presence and density within myocardium could create those attractive premises of new therapeutic targets for heart regeneration/repair after MI, or remodeling in HF.

PI3K/AKT gain of function would upregulate the proliferation of cardiomyocytes [148]. PI3K/Akt pathway promotes cell survival through the inhibition of apoptosis. Moreover, in the pathogeny of MI, the PI3K-AKT pathway has an anti-apoptotic effect [149]. This effect would be explained by translocation of phospho-AKT (pAKT) with secondary modification of cytochrome c oxidase (CcO) activity [138]. Thus, upregulating PI3K/Akt-p53 signalling pathway could offer cardioprotection in MI injury.

Under these circumstances, it is tempting to believe that TCs (cells of mesenchymal origin), such as progenitor cells or nursing cells, could represent the missing link or those novel targets for cellular treatment of several cardiac disorders, including MI or ischemic HF. However, any remarkable difficulties in delivering TCs into myocardium would be represented by the method of delivery and also the survival of grafted cells. These hinderances could be represented by the absence of paracrine factors, tissue guidance, blood vessel scaffolds and/or neural inputs.

Nevertheless, an attractive solution is to potentiate endogenous cardiac repair after MI by stimulating the cooperation between TCs and CSCs. In other words, the tandem TCs–CSCs could be a better option for therapy, rather than CSCs alone. Heart TCs may have the potential to function after grafting in myocardium with presumptive important advantages regarding availability and plasticity of CSCs.

## Data Availability

Not applicable.
